# Sex- and mouse strain-related differences in body weight gain, composition of the gut microbiota, and levels of selected metabolites in response to a Western-style diet

**DOI:** 10.1186/s12876-026-04647-2

**Published:** 2026-02-04

**Authors:** Katarzyna Unrug-Bielawska, Monika Dziełak, Zuzanna Sandowska-Markiewicz, Magdalena Piątkowska, Paweł Czarnowski, Krzysztof Goryca, Natalia Zeber-Lubecka, Michalina Dąbrowska, Aneta Bałabas, Małgorzata Statkiewicz, Izabela Rumieńczyk, Kazimiera Pyśniak, Urszula Wójcik-Trechcińska, Anita Tyl-Bielicka, Joanna Ziemska-Legięcka, Michał Mikula, Jerzy Ostrowski

**Affiliations:** 1 Department of Genetics, Maria Sklodowska-Curie National Research Institute of Oncology, Warsaw, Poland; 2 Department of Gastroenterology, Hepatology and Clinical Oncology, Centre of Postgraduate Medical Education, Warsaw, Poland

**Keywords:** Western diet, GAN diet, MTARC knockout mice, Stool microbiota, Stool metabolites

## Abstract

**Background:**

Recent studies reveal an association between the mitochondrial Amidoxime Reducing Component (MTARC) 1 and 2 proteins and metabolism in Mtarc1/2-deficient mice that are resistant to diet-induced obesity; however, the impact of Mtarc1/2 knockout (KO) on the gut microbiota and metabolome has not been explored in the context of sex and diet.

**Aim:**

To compare the effects of a Western diet (WD) or a Novel Gubra Amylin NASH (GAN) diet on body weight gain, and on the composition of the gut microbiome and metabolome, between the background mouse strain and male and female Mtarc1- or Mtarc2-KO mice.

**Methods:**

Seventy-two 8-week-old male and female mice from each strain were fed a WD or a corresponding control normal diet (ND/WD), or a GAN diet or a corresponding control normal diet (ND/GAN), for 16 weeks. Fecal samples were collected at the beginning and end of the experiments, and 16 S rRNA-based microbiota profiling-based analysis was performed by sequencing the variable V3 and V4 regions of the bacterial 16 S rRNA gene. Mass spectrometry was used to measure short-chain fatty acids (SCFAs) and amino acids (AAs).

**Results:**

Compared with a control ND, GAN feeding increased the body weight of all groups of mice, whereas the WD increased the body weight of all groups except Mtarc2-KO female mice. The most significant weight gain was observed for male and female C57BL6/NTac mice fed a WD or GAN. Differences in body weight were mirrored in the microbiota profiles. In each of the mouse strains tested, the number of differentially abundant taxa between the GAN- and ND/GAN-fed groups was greater than that between WD- and ND/WD-fed mice. Both the GAN and WD also altered the levels of SCFAs and AAs in feces in a manner dependent on the mouse strain and sex.

**Conclusions:**

Significant differences in body weight gain and changes in the composition of the gut microbiome and metabolome between the background mouse strain and Mtarc1-KO or Mtarc2-KO mice were further modified by sex and diet. Therefore, preclinical studies using animal models of obesity should ensure the selection of the appropriate mouse strain and sex, and be mindful of diet composition.

**Supplementary Information:**

The online version contains supplementary material available at 10.1186/s12876-026-04647-2.

## Background

The mitochondrial Amidoxime Reducing Component (MTARC) complex, comprising MTARC1/2, cytochrome b5 (CYB5B), and cytochrome b5 reductase (CYB5R), metabolizes a large variety of N-hydroxylated compounds [1, 2]. More recently, it has been associated with lipid metabolism [3]. Previously, we showed that expression of Mtarc1 and 2, as well as the N-reductive activity of the complex, in the murine liver decreased under fasting conditions, and increased after exposure to a high-fat diet (HFD) [4]. We also showed that mice lacking the Mtarc2 gene (Mtarc2-KO) are resistant to HFD-induced obesity [5]. More recently, genome-wide association studies (GWAS) identified protective MTARC1 variants such as p.A165T, as well as loss-of-function mutations, that reduce plasma lipids, liver triglycerides, and liver-related mortality in patients with metabolic dysfunction-associated steatotic liver disease (MASLD) [6–8]. Research into the MTARC1 gene in preclinical models shows that targeting Mtrac1 via siRNA or genetic modulation improves MASLD outcomes by reducing liver steatosis, fibrosis markers, and oxidative stress [9, 10]; however, the precise molecular functions (i.e., enzymatic activity, protein stability, or lipid transport) remain unclear.

The composition of the gut microbiota is affected by host genotype, age, and sex, and is highly associated with changes in diet and physical activity, both of which are the main modulators of the gut microbiota-host metabolism axis [11, 12]. Both high-fat and high-fructose diets cause imbalances in the intestinal microbiota (i.e., dysbiosis) that can affect a complex network of host-microbe interactions [13–18]; however, there is still no definitive answer to the question of how changes in the gut microbiome affect the development of obesity. Enterocytes, which line the intestinal epithelium, are central to the lipid digestion, absorption, and transport, processes that are intricately modulated by microbial metabolites and enzymatic activity. Commensal bacteria such as Bacteroides and Prevotella synthesize sphingolipids that integrate into host cell membranes, thereby influencing intestinal inflammation and ceramide pools [19]. Additionally, the gut microbiota deconjugates and modifies primary bile acids to generate secondary forms that regulate enterocyte lipid absorption by modulating bile acid signaling pathways and emulsification efficiency, which in turn have a direct impact on cholesterol and triglyceride uptake [20]. Finally, bacteria such as *Lactobacillus* and *Bifidobacterium* produce conjugated fatty acids and hydroxylated fatty acids that alter lipid storage by enterocytes, as well as lipoprotein assembly, thereby affecting systemic lipid levels [21].

Given the emerging role of the MTARC complex in lipid metabolism, and the lack of studies investigating its molecular function in connection with the gut microbiota, we used male and female Mtarc1-KO and Mtarc2-KO mice to examine the impact of two Western-style diets, a high-fat/high-sucrose (Western Diet (WD)) and high-fat/high-fructose/high-cholesterol (GAN) diet on weight gain, the composition of the intestinal microbiota, and levels of selected stool metabolites (short-chain fatty acids (SCFAs) and amino acids (AAs)). As expected, sexual dimorphism appeared to be the main factor underlying changes in the parameters tested.

## Materials and methods

### Mice

All experiments involving animals were approved by the Local Ethics Committee (decisions: WAW2/119/2019, WAW2/098/2021 and WAW2/124/2021). All studies were carried out according to the European Parliament and the Council Directive (2010/63/EU) and the Polish regulations on the protection of animals used for scientific and educational purposes (Journal of Laws 2021, items 1331 and 2338).

Mtarc1-KO and Mtarc2-KO (C57BL/6NTac Mtarc1/Mtarc2-KO) mice and the background strain (C57BL/6NTac B6) mice were born in a core specific pathogen-free facility at the Maria Sklodowska-Curie National Research Institute of Oncology, Warsaw. The genotype of all mice used in the experiments was verified prior to selection, in accordance with standard procedures. Mice were kept under standard humidity (55% ± 10%) and temperature (21 ± 2 °C) conditions in climate-controlled rooms under a 12/12 h light/dark cycle. Animals were tested for the presence of viruses, bacteria, and parasites in accordance with the recommendations of the Federation of European Laboratory Animal Science Associations.

Thirty-six male and 36 female mice (8 weeks old) of each strain were assigned randomly to one of the following diet groups: a high-fat/high-sucrose diet (WD), a corresponding control normal diet (ND/WD), a high-fat/high-fructose/high-cholesterol diet (Gubra Amylin NASH [GAN] diet), or a corresponding control ND (ND/GAN). Mice were randomly assigned to cages and experimental groups to avoid systematic bias; all animals were housed under identical environmental conditions, and sample processing was randomized to minimize cage and batch effects. Mice were fed these diets for 16 weeks. The composition of each diet is shown in Table 1. Animals had unrestricted access to water and food throughout the experiment. Stool samples were collected from each mouse before, and at the end of, the 16-week experiment and stored at − 80 °C until use. At the end of the experiment, mice were anesthetized with 5% isoflurane and sacrificed by cervical dislocation.

**Table 1:** Composition of the WD (Western diet D12079B) and its corresponding Chow normal diet (ND/WD; D14042701N), and of the GAN diet (GAN D091000310) and its corresponding Chow normal diet (ND/GAN; D09100304)

Ingredient, gram	WD D12079B	ND/WD D14042701N	GAN D091000310	ND/GAN D09100304
Caseine	195	195	200	200
Methionine	3	3		
L-Cystine			3	3
Sucrose	350		100	100
Fructose			200	
Maltodextrin	100	150	100	85
Dextrose				169
Corn Starch	50	695		350
Soybean Oil			25	25
Lard			20	20
Butter	200	42.5		
Corn Oil	10	10		
Palm Oil			135	
Cholesterol	1,5		18	
Fiber (Cellulose)	50	50	50	50
Minerals	39	39	50	50
Vitamins	3	12	3	3
Other (Dyes, Antioxidants)	0.04	0.04	0.06	0.06
Total	1001.54	1196.54	904.06	1055.06

The composition of each of the diets was as follows: The WD comprised 17% protein, 43% carbohydrate, and 40% fat, whereas the corresponding control ND/WD diet comprised 17% protein, 73% carbohydrate, and 10% fat. The GAN diet comprised 20% protein, 40% carbohydrate, and 40% fat, and its corresponding control ND/GAN diet comprised 20% protein, 70% carbohydrate, and 10% fat. Butter was the primary fat source in the WD, and was also used as the fat source in the control ND/WD (at ¼ of the amount). The primary fat source in the GAN diet was palm oil, with added soybean oil and lard, while that in the control ND/GAN was soybean oil and lard (at the exact amounts as in the GAN). The primary carbohydrate source in the WD was sucrose, and that in the control ND/WD was corn starch, although the ND/WD also contained some sucrose (at 1/3 of the amount in the WD). The main carbohydrate component in GAN was fructose, whereas that in the control ND/GAN was corn starch, along with some dextrose (Table 1). Both the GAN and WD diets contained cholesterol (2% and 0.1% (by weight), respectively).

### DNA analysis

Genomic DNA was isolated from fecal samples using a QIAamp Fast DNA Stool Mini Kit (Qiagen, Hilden, Germany) as previously described [22]. The quality and quantity of the extracted DNA was assessed by measuring the optical density in a NanoDrop 2000/2000c spectrophotometer (Thermo Fisher Scientific, Carlsbad, CA, USA) and fluorometrically using a Qubit dsDNA HS Assay Kit (Thermo Fisher Scientific). Preparation of the variable V3 and V4 region libraries from the 16 S bacterial 16 S rRNA gene was carried out following the 16 S Metagenomic Sequencing Library Preparation protocol on an Illumina platform (Illumina, Inc., San Diego, CA, USA). Sequences were obtained by an Illumina MiSeq system, with paired-end reads of 2 × 300 bp.

### Metabolite analysis

SCFAs and AAs were extracted and derivatized as previously described [22–24]. Gas chromatographic analysis of fecal extracts was performed on an Agilent 7000D triple quadrupole mass spectrometer coupled to a 7890 gas chromatography system with a G4513A autosampler (Agilent Technologies, Santa Clara, CA, USA). A VF-5ms column (30 m, 0.25 mm, 0.50 μm) was used for analysis. Mass spectrometry data were collected in full-scan mode for m/z 15–650, at a frequency of 4.9 scans per second. Data were analyzed using MassHunter software (Agilent Technologies).

### Statistical analysis

The DADA2 [25] pipeline version 1.30 was used for read error correction, amplicon sequence variant identification, and chimeric read identification and removal. Taxonomy assignment was carried out with DECIPHER version 2.30 [26] using SILVA [version 1.138 [27]. Diversity indexes (Shannon and Chao1) were calculated using the phyloseq R package (version 1.46) [28]. Significant differences between groups were calculated by the DEseq2 package. Distances (β-diversity) were calculated by the phyloseq package [28] using the Euclidean metric. Spearman’s rank correlation coefficients have been calculated in R (R version: 4.1.2) with stats package (version: 4.1.2) between metabolites and the participation of bacteria in groups of mice defined by strain, sex and diet. The calculation of correlations was achieved by the calculation of normalised numbers of bacteria reads in each probe. The results of this study have been evaluated to identify statistically significant outcomes (i.e. p-values < 0.1) that exhibited strong intercorrelations (*r* > 0.6 or *r* < − 0.6). The remaining correlations have been assigned a value of 0 in the corresponding plots. Heatmaps were generated using the R package pheatmap (version 1.0.13) with the Ward.D method and Euclidean distance bacteria clustering [29]. The difference in the body weights and relative abundance of AAs and SCFAs was determined by a two-way ANOVA in GraphPad Prism v10.1.1.

## Results

### Effects of diet on body weight

**Fig. 1:**
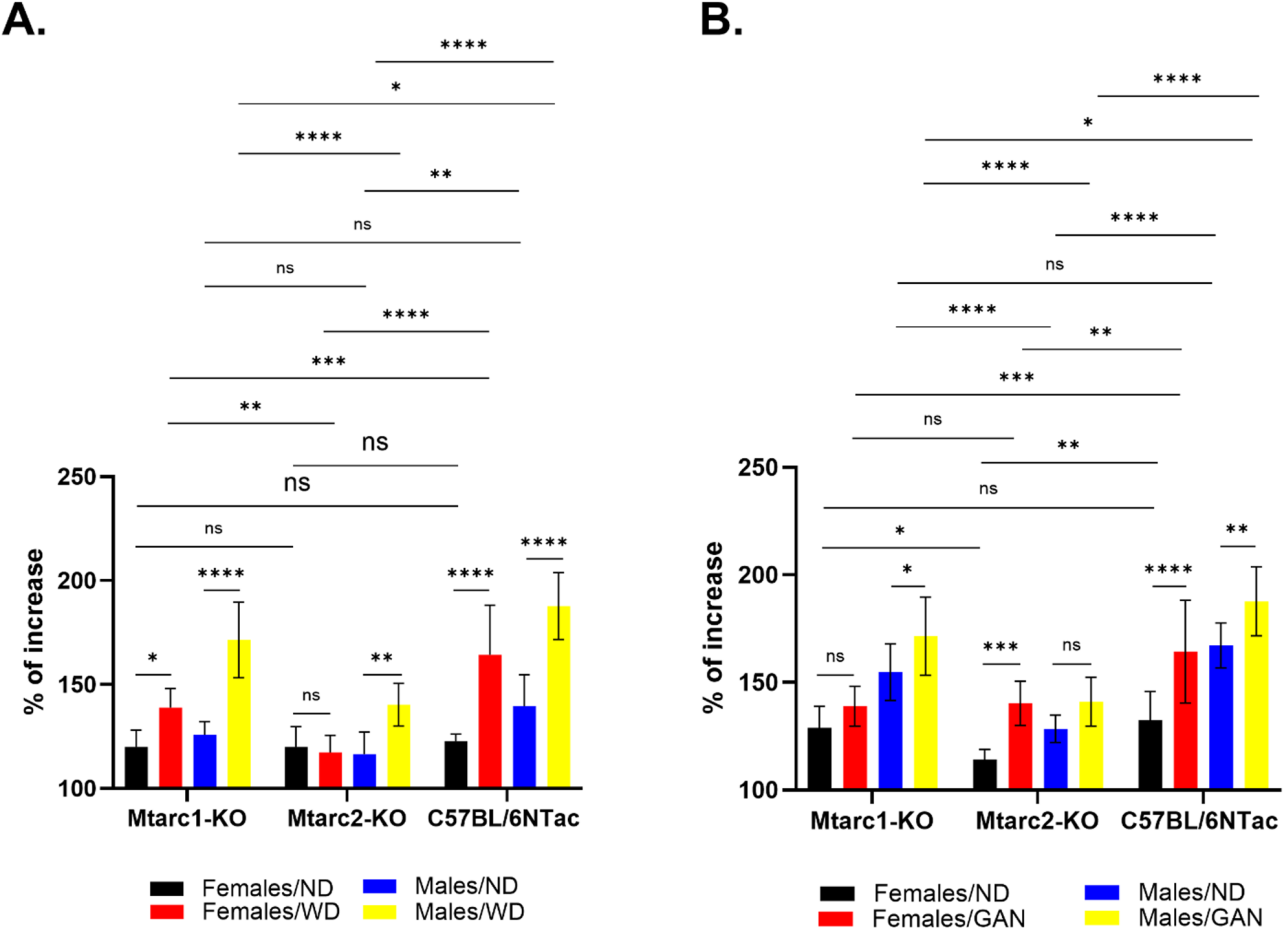
Body weight change at week 16 by Mtarc1-KO, Mtarc2-KO, and C57BL/6NTac mice fed a high-fat/high-sucrose diet (WD) (**A**) or a high-fat/high-fructose/high-cholesterol diet (GAN) (**B**). Data are presented as the mean ± SD. Asterisks indicate significant differences; * adjp < 0.05, ** adjp < 0.01, *** adjp < 0.001, **** adjp < 0.0001 (statistical assessment is conducted using a two-way ANOVA; 8–10 mice per a group)

The animals (8–10 mice per group) were fed obesogenic or corresponding ND diets for 16 weeks. To determine how the body weight at the end of the experiments was affected by diets, a two-way ANOVA test was conducted. Weight gain of Mtarc1-KO, Mtarc2-KO, and C57BL6/NTac female and male mice groups fed the control ND/WD was similar (Fig. 1A), whereas Mtarc2-KO females and males fed the control ND/GAN achieved significantly lower weight gain than the corresponding mouse groups of the two other strains (Fig. 1B). The body weight of all groups of mice fed the WD, exept for female Mtarc2-KO mice, was higher than that of mice fed the corresponding control ND/GAN. A similar increase in body weight was observed in GAN-fed mice, except Mtarc1-KO females. Male and female C57BL6/NTac mice fed a WD or a GAN diet showed the greatest increases in body weight, whereas the male Mtarc2-KO mice fed a WD or GAN diet, respectively, showed the smallest growth in body weight at the end of the feeding period (Figs. 1A and B).

**Fig. 2:**
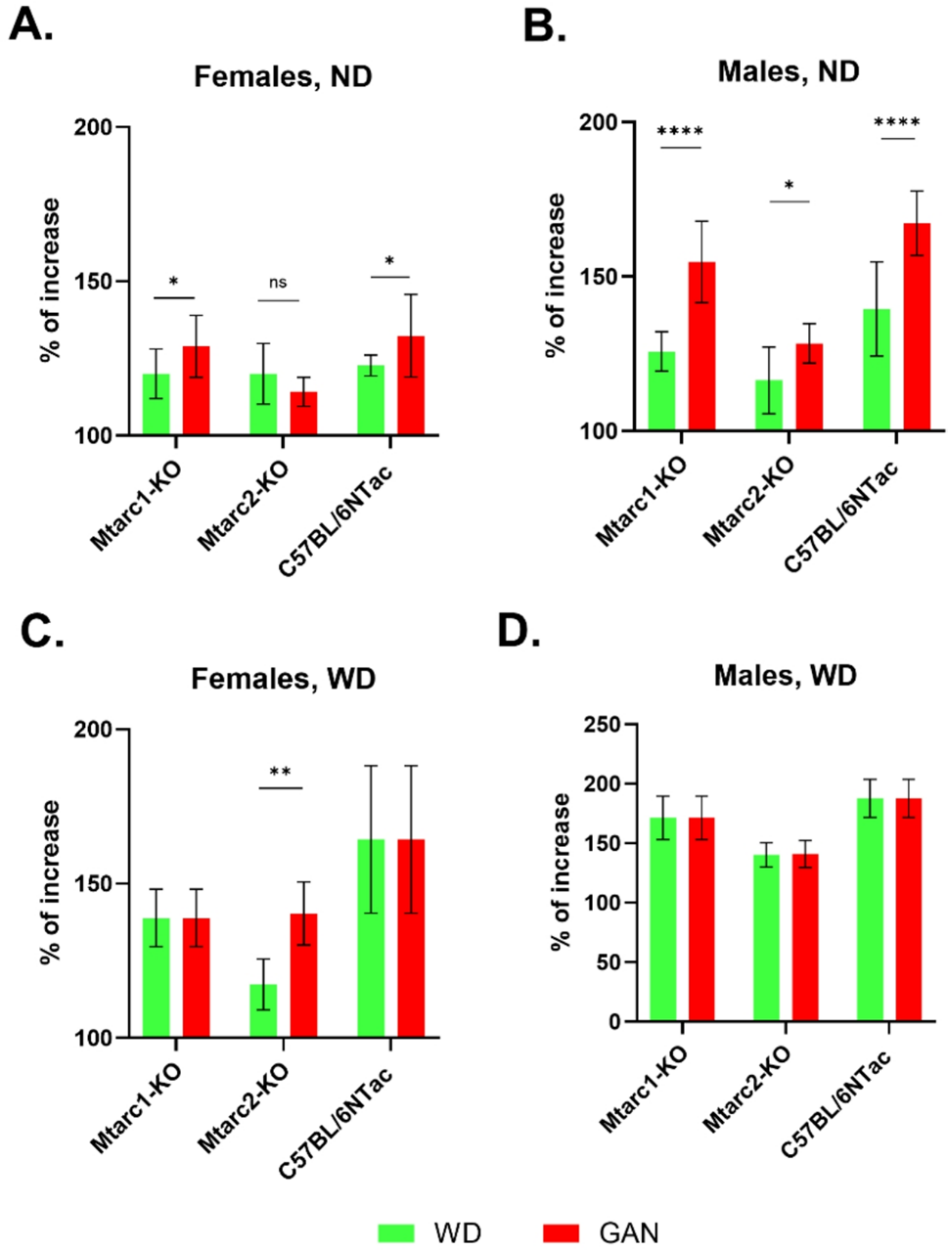
Comparison of the body weight of Mtarc1, Mtarc2, and C57BL/6NTac mice fed a control ND/WD and ND/GAN diets (**A** and **B**), or a WD or GAN diets (**C** and **D**). Data are presented as the mean ± SD. Asterisks indicate significant differences; * adjp < 0.05, ** adjp < 0.01, **** adjp < 0.0001 (statistical assessment is conducted using a two-way ANOVA; 8–10 mice per group)

We found significantly greater weight gain in males of all three strains and female Mtarc1-KO and C57BL6/NTac mice fed ND/GAN than ND/WD. (Figure 2A and B). Feeding with GAN resulted in higher body weight only in Mtarc2-KO females than in those fed with WD (Fig. 2C and D).

### Effect of different diets on the composition of the gut microbiota and metabolite profiles

Next, we analyzed the α- and β-diversity of the fecal microbiota. α-diversity was analyzed using the Shannon index (a marker of bacterial richness and evenness) and the Chao1 index (a marker of bacterial richness). β-diversity was analyzed using principal coordinate analysis of Euclidean distance. All analyses were performed at the genus level.

**Fig. 3:**
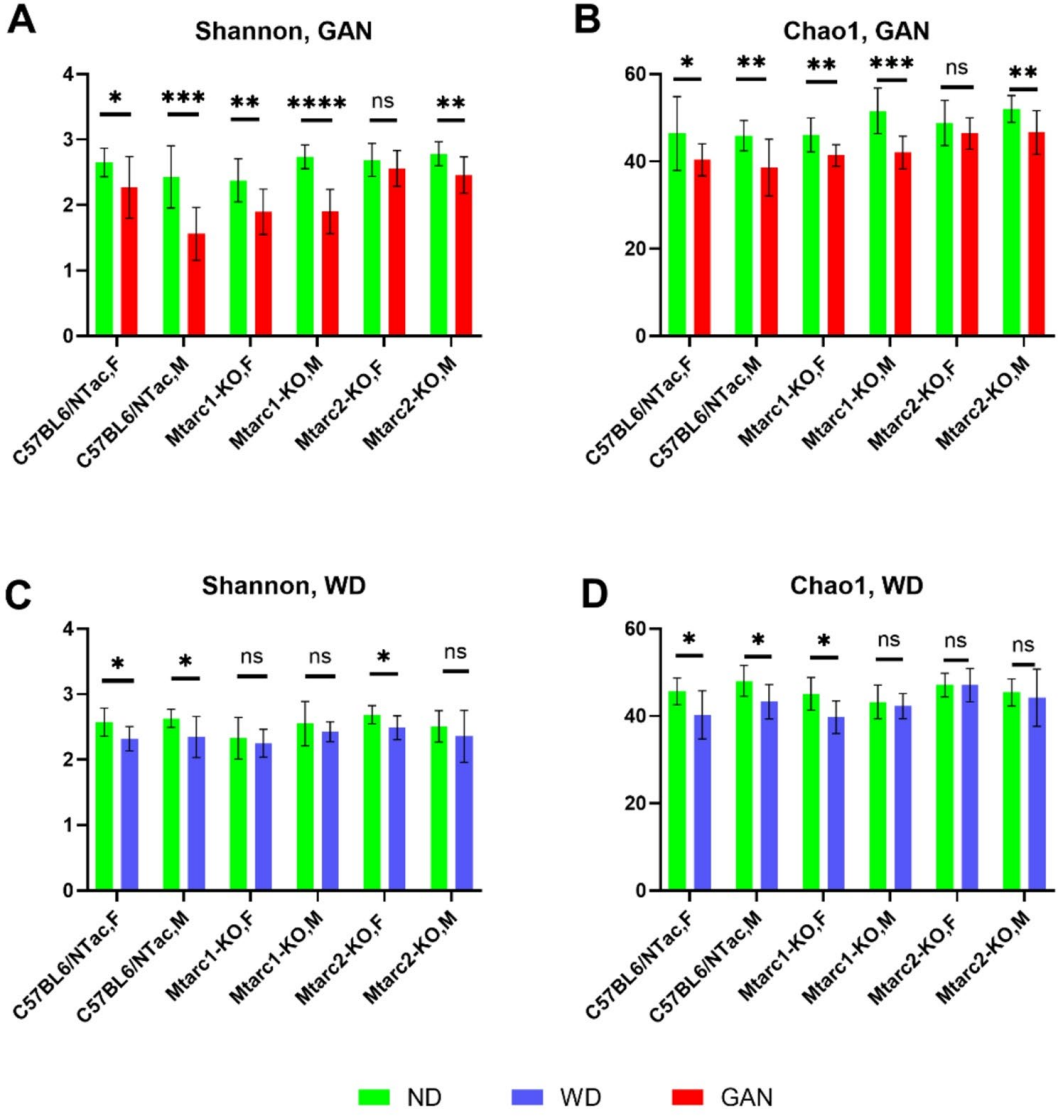
α-diversity (analyzed by the Shannon and Chao1 indices) in fecal samples collected after the 16th week of feeding. Data are presented as the mean ± SD. Asterisks indicate significant differences; * *p* < 0.05, ** *p* < 0.01, *** *p* < 0.001, **** *p* < 0.0001 (unpaired two-tailed Student’s t-test; 8–10 mice per group)

After correcting for multiple hypothesis testing, the results showed that 16 weeks of GAN feeding decreased the Shannon (Fig. 3A) and Chao1 (Fig. 3B) indices in all groups of mice except female Mtarc2-KO mice when compared with mice fed the corresponding ND. The WD decreased the Shannon index in C57BL/6NTac females and males, and in Mtarc2-KO females (Fig. 3C), and decreased the Chao1 index in C57BL/6NTac females and males, and in Mtarc1-KO females (Fig. 3D).

**Fig. 4:**
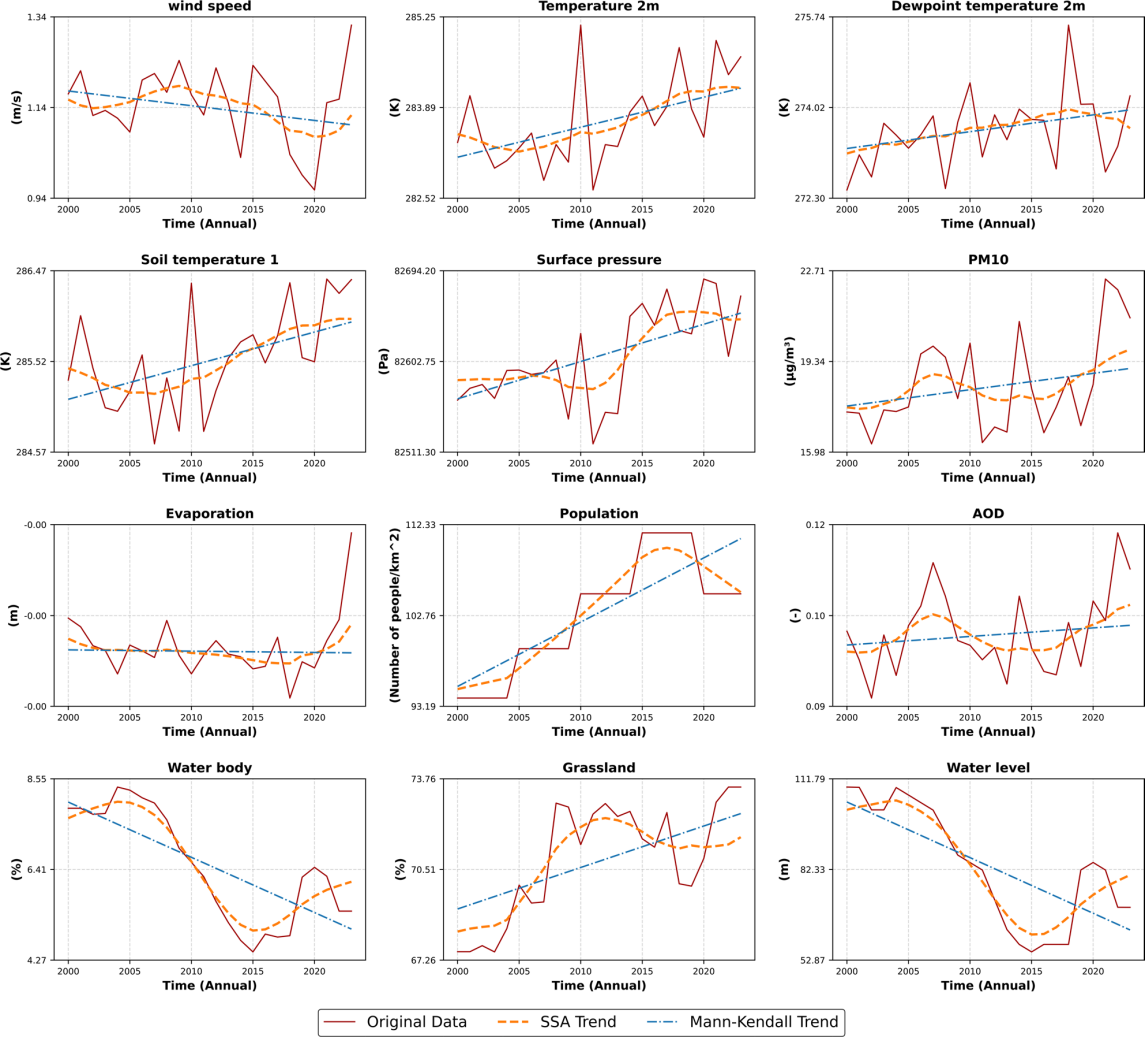
Principal coordinate analysis (PCoA) using the Euclidean metric of fecal samples collected at the end of the experiment. Each dot represents a single sample

Feeding a GAN (Fig. 4A) or WD (Fig. 4B) for 16 weeks changed β-diversity of the microbial community, as measured by principal coordinate analysis (PCoA), compared with that in the corresponding control groups fed a ND. The significance of the differences between the groups was tested using t-tests (Table 2).

**Table 2:** Statistical significance of the differences in β-diversity (measured by principal coordinate analysis for the first (PCoA1) and second (PCoA2) components) of the fecal microbiota of mice fed a high-fat diet/high-sucrose (WD), a high-fructose/high-cholesterol diet (GAN), or a corresponding control Chow control diet (ND)

GAN vs. corresponding ND	PCoA1	PCoA2
C57BL/6NTac females	1,42E-07	0.333265
C57BL/6NTac males	5,22E-06	0.263387
Mtarc1-KO females	3,88E-06	0.15833
Mtarc1-KO males	1,57E-07	0.212444
Mtarc2-KO females	3,98E-08	0.229111
Mtarc2-KO males	6,35E-06	0.363483

WD vs. corresponding ND	PCoA1	PCoA2
C57BL/6NTac females	0,001387	0.328018
C57BL/6NTac males	0,001147	0.425827
Mtarc1-KO females	1,75E-05	0.226287
Mtarc1-KO males	1,46E-05	0.227403
Mtarc2-KO females	0,012322	0.974545
Mtarc2-KO males	0,025603	0.494122

Taxonomic analysis of fecal samples collected after 16 weeks of GAN feeding identified 26 (13 over-represented), 29 (11 over-represented) and eight (five over-represented) genera showing significant differences in abundance (adjusted *p* < 0.05) in C57BL/6NTac, Mtarc1-KO, and Mtarc2-KO females, respectively, and 25 (nine over-represented), 25 (11 over-represented) and 15 (six over-represented) genera showing significant differences in abundance in C57BL/6NTac, Mtarc1-KO, and Mtarc2-KO males, respectively, compared with mice fed the corresponding control ND (Supplementary Table 1). A similar analysis of WD-fed mice identified 12 (six over-represented), eight (one over-represented), and one genera showing significant differences in abundance in C57BL/6NTac, Mtarc1-KO, and Mtarc2-KO females, respectively, and seven (five over-represented), 19 (eight over-represented), and two (one over-represented) showing significant differences in abundance in C57BL/6NTac, Mtarc1-KO, and Mtarc2-KO males, respectively (Supplementary Table 2).

**Fig. 5:**
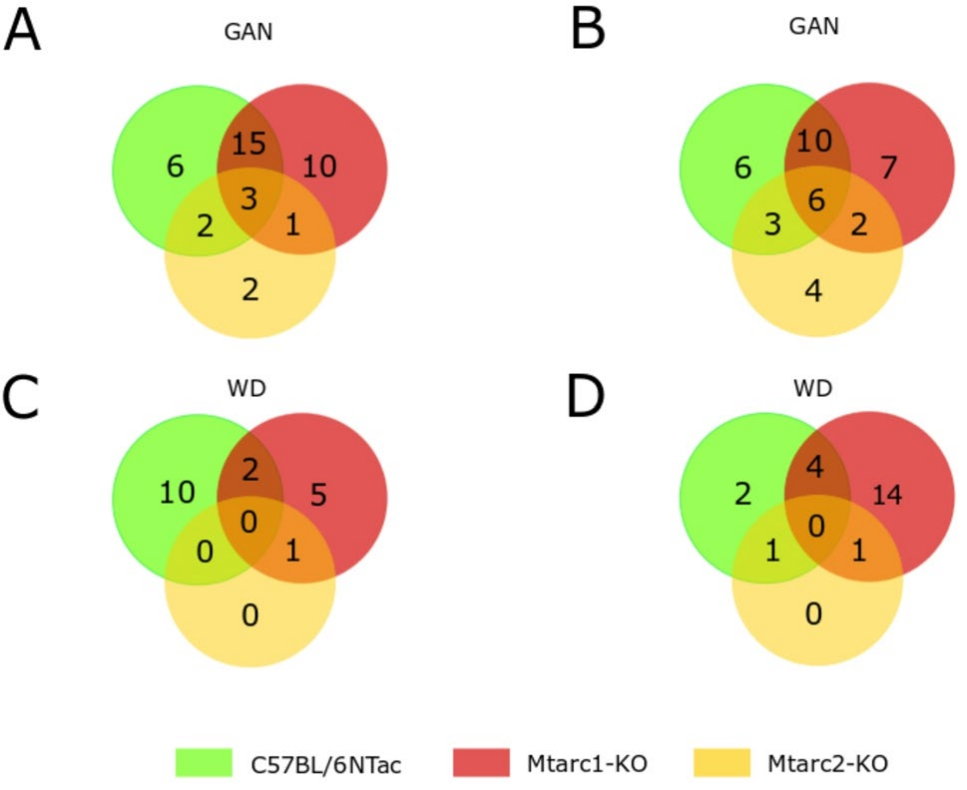
Number of genera showing a change in relative abundance in high-fed/high-fructose/high-cholesterol diet (GAN)-fed, and high-fat diet/high-sucrose (WD)-fed female and male C57BL/6NTac, Mtarc1-KO, and Mtarc2-KO mice (compared with the same strains of mice fed the corresponding normal diet (ND))

Separate Venn diagrams generated for females and males fed a GAN or WD demonstrated that the number of differentially abundant taxa in GAN-fed mice was higher than that in WD-fed mice. In addition, in both sexes of Mtarc2-KO mice, the number of differentially expressed bacteria after feeding a GAN or WD was lower than that in C57BL/6NTac and Mtarc1-KO mice (Fig. 5). One over-represented genus (*Lachnoclostridium*) and two under-represented genera ([Eubacterium] *brachy group*, *Paludicola*) were shared by all female groups fed a GAN diet. One over-represented genus ([Eubacterium] brachy group) and five under-represented genera (*Bifidobacterium*,* Ileibacterium*,* Lachnospiraceae UCG-008*,* Roseburia*,* Paludicola*) were shared by all male mice fed a GAN diet (Fig. 5).

Fifteen genera (four over-represented: *Parabacteroides*,* ASF356*,* Bilophila*, and *Mucispirillum*; and 11 under-represented: *Candidatus Saccharimonas*,* Dubosiella*,* Anaerotruncus*,* Lachnospiraceae NK4A136 group*, [Eubacterium] *oxidoreducens group*,* Turicibacter*,* Clostridium sensu stricto 1*,* Faecalibaculum*, [Eubacterium] *nodatum group*,* Marvinbryantia*, and *Defluviitaleaceae UCG-011*), and 10 genera (five over-represented: *Intestinimonas*,* Anaerotruncus*,* Tuzzerella*,* Helicobacter*, and *Blautia*; and five under-represented: *Candidatus Saccharimonas*,* Akkermansia*,* Romboutsia*,* Clostridium sensu stricto 1*,* and Defluviitaleaceae UCG-011*) showed altered abundance in GAN-fed C57BL/6NTac and Mtarc1-KO females and males, respectively, when compared with the respective control groups fed a control ND.

**Fig. 6:**
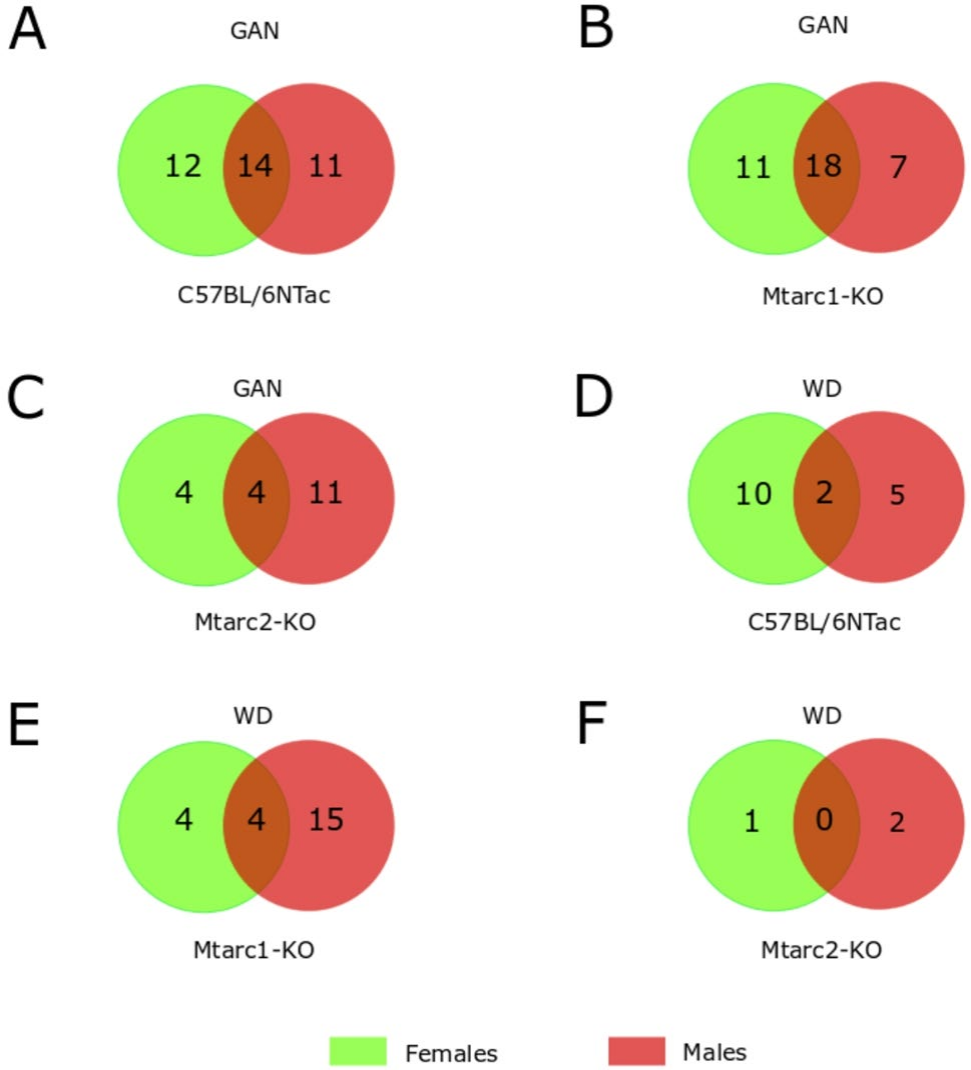
Number of genera showing differences in relative abundance in female and male C57BL/6NTac, Mtarc1-KO, and Mtarc2-KO mice fed a high-fat/high-fructose/high-cholesterol (GAN) diet or a high-fat/high-sucrose (WD) diet

When we compared the number of differential taxa between females and males of different strains fed a GAN or WD, we found significant differences in the number of commonly altered taxa between individual groups. The most commonly altered taxa were found in GAN-fed female C57BL/6NTac and Mtarc1-KO mice; there were clearly fewer commonly altered taxa in all other groups (Fig. 6).

**Fig. 7:**
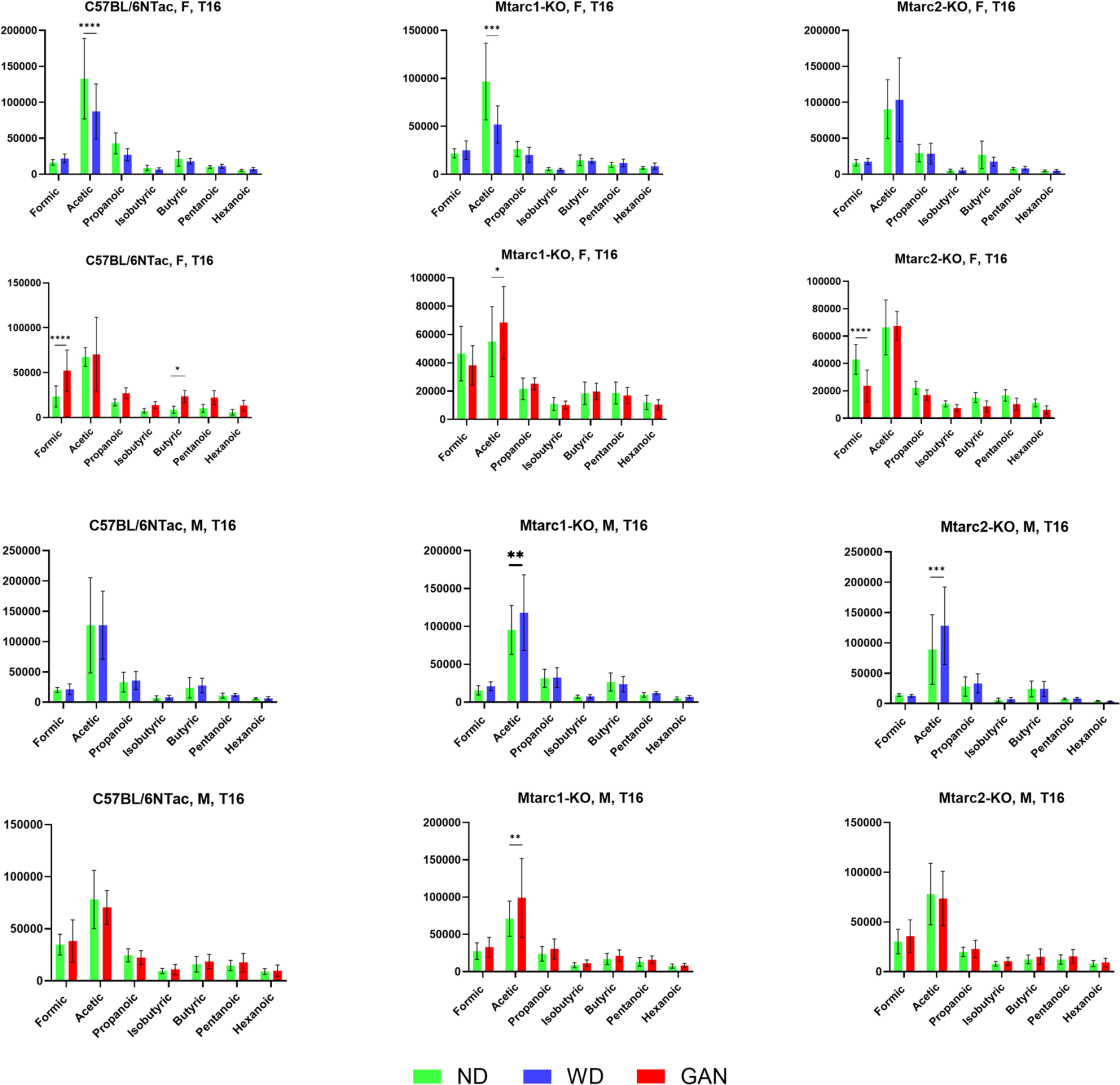
Differences in the relative abundance of short-chain fatty acids (SCFAs) between C57BL/6NTac, Mtarc1-KO, and Mtarc2-KO mice fed a high-fat/high-sucrose diet (WD) or a high-fat/high-fructose/high-cholesterol diet (GAN) for 16 weeks, and those in mice fed the corresponding normal diet (ND). Data are presented as the mean ± SD. Asterisks indicate significant differences: * *p* < 0.05, ** *p* < 0.01, *** *p* < 0.001, **** *p* < 0.0001 (the two-way ANOVA; 8–10 mice per group)

Gas chromatography/mass spectrometry-based analyses identified seven SCFAs (formic acid, acetic acid, propanoic acid, isobutyric acid, butanoic acid, pentanoic acid, and hexanoic acid) and nine AAs (alanine, glycine, valine, leucine, isoleucine, proline, methionine, phenylalanine and tyrosine) in fecal sample extracts. Pairwise comparisons between the study groups, using the two-way ANOVA, revealed increases in the relative abundance of formic and butyric acids in GAN-fed C57BL/6NTac females and acetic acid in GAN-fed Mtarc1-KO females, in WD- and GAN-fed Mtarc1-KO males, and WD-fed Mtarc2-KO males compared with the same strains fed the corresponding control ND. In turn, a decrease in the abundance of formic acid was observed in GAN-fed Mtarc2-KO females and of acetic acid – in WD-fed C57BL/6NTac and Mtarc1-KO females (Fig. 7).

A comparison of relative SCFAs levels between the three strains revealed differences of acetic acid levels between females fed the ND/WD, ND/GAN and WD, of formic acid between females fed the ND/GAN and the GAN, and of butyric acid between female C57BL/6NTac and Mtarc2-KO mice fed the GAN diet. The comparison between male groups showed differences in acetic acid levels between males fed ND-WD and those fed GAN (Supplementary Fig. 1).

**Fig. 8:**
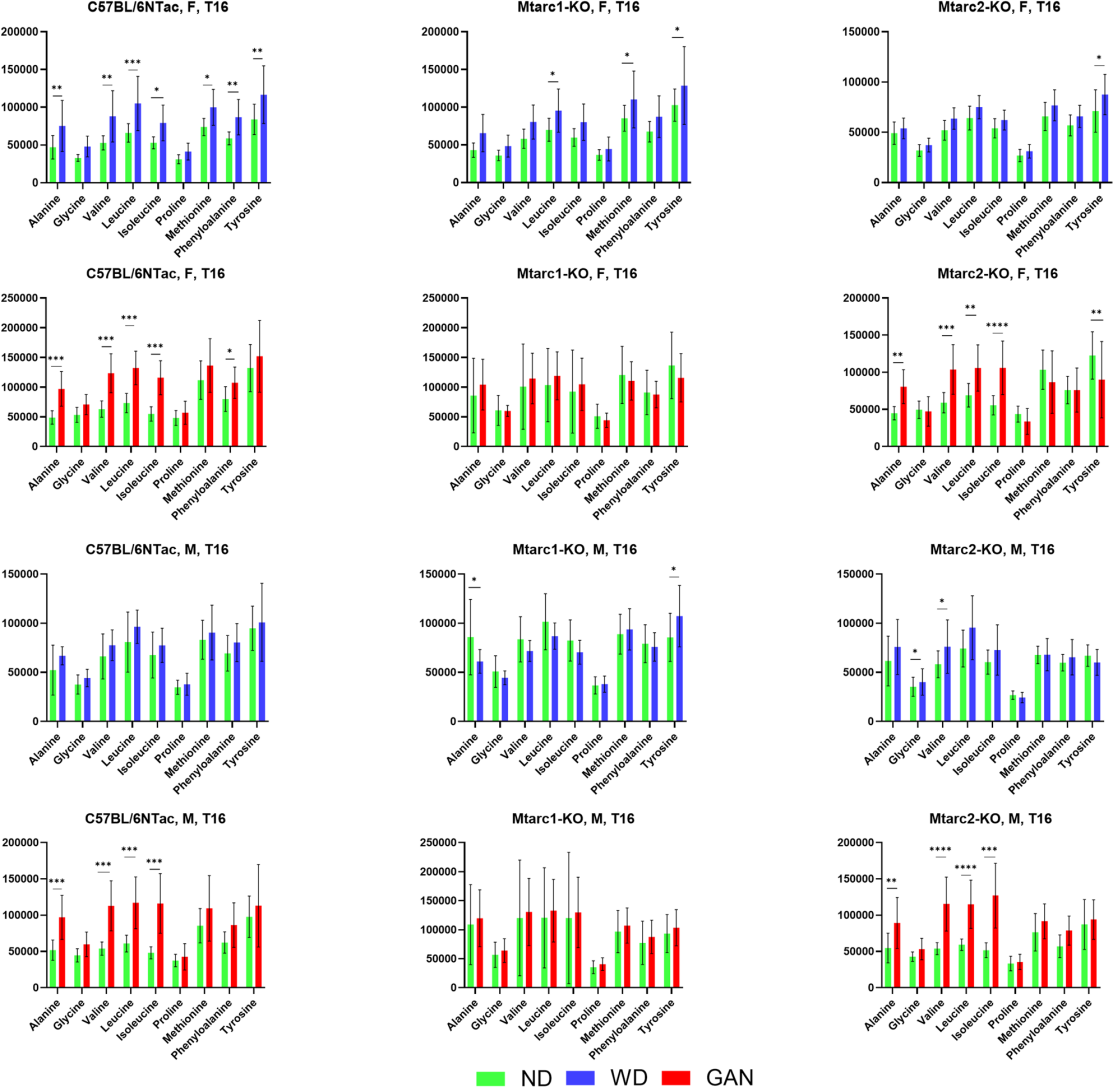
Differences in the relative abundance of short-chain fatty acids (SCFAs) between C57BL/6NTac, Mtarc1-KO, and Mtarc2-KO mice fed a high-fat/high-sucrose diet (WD) or a high-fat/high-fructose/high-cholesterol diet (GAN) for 16 weeks, and that in those fed the corresponding normal diet (ND). Data are presented as the mean ± SD. Asterisks indicate significant differences; * *p* < 0.05, ** *p* < 0.01, *** *p* < 0.001, **** *p* < 0.0001 (the two-way ANOVA, 8–10 mice per group)

Compared with the corresponding control ND, the WD led to a significant increase in the abundance of seven out of nine (alanine, valine, leucine, isoleucine, methionine, phenylalanine and tyrosine) and five AAs (alanine, valine, leucine, isoleucine, and phenylalanine) in C57BL/6NTac females, three (leucine, methionine, and tyrosine) and none AAs in Mtarc1-KO females, and one (tyrosine) and five (alanine, valine, leucine, isoleucine, and tyrosine) in Mtarc2-KO females fed WD and GAN, respectively. Similar comparisons conducted in male groups showed significant increase in the abundance od none AAs and four AAs (alanine, valine, leucine, and isoleucine) in C57BL/6NTac males, three AAs (leucine, methionine, and tyrosine) and none AAs in Mtarc1-KO males, two (glycine and valine) and four AAs (alanine, valine, leucine, and isoleucine) in Mtarc2-KO males fed WD and GAN, respectively (Fig. 8). Again, we found significant differences in the relative abundance of rather limited number of AAs between groups of mice that were related to sex and diet. Most of the differences in abundance occurred between males fed WD and GAN diets compared with the corresponding ND-fed controls (Supplementary Fig. 2).

### The correlation analysis of identified bacterial taxa and metabolites

**Fig. 9:**
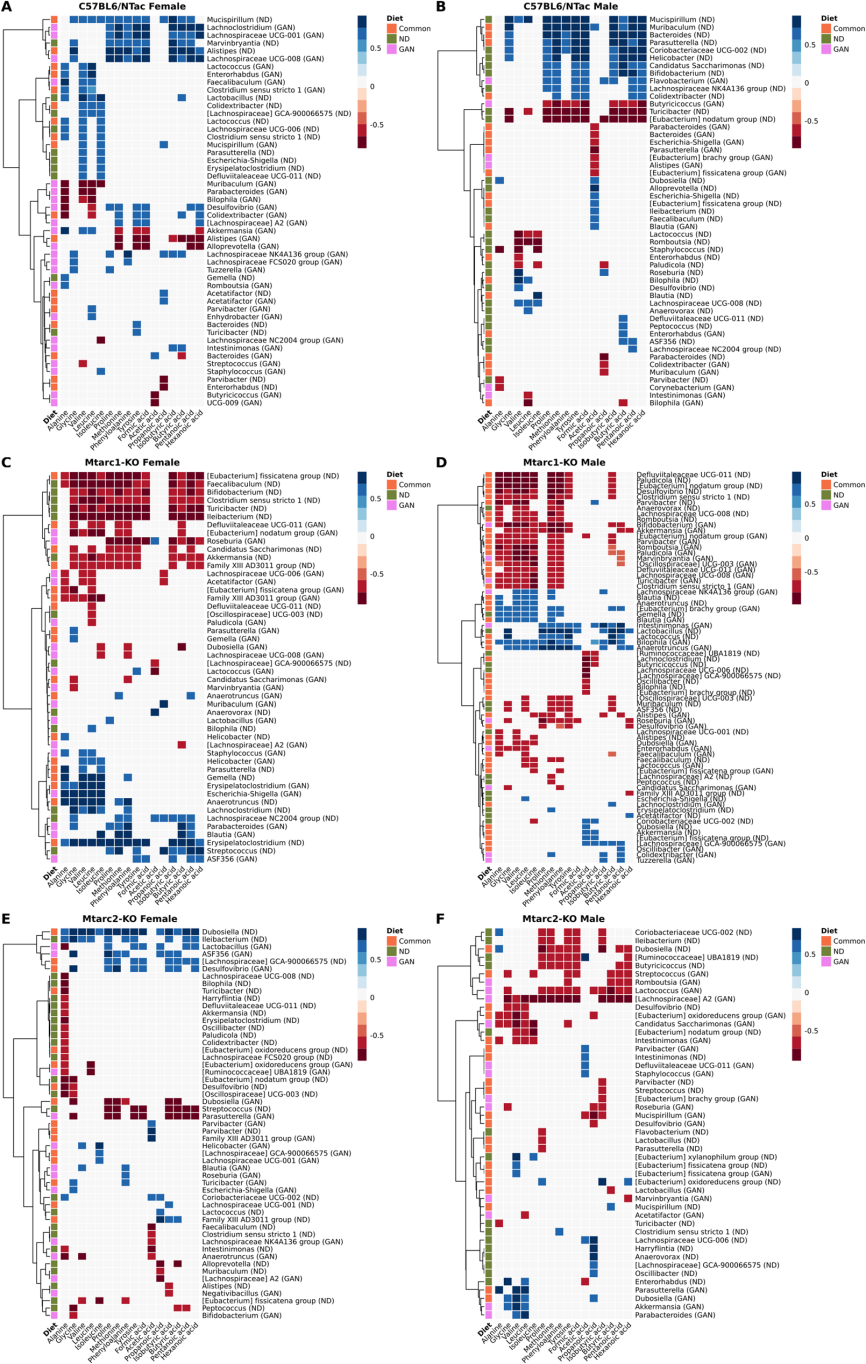
Correlation coefficients between bacterial taxa and amino acids (AAs) and short-chain fatty acids (SCFAs) in C57BL6/NTac, Mtarc1-KO, and Mtarc2-KO female and male mice groups fed GAN and the control ND/GAN diets. Orange squares indicate correlations common to both diets, green squares are unique to the ND diet, and pink squares are unique to the GAN diet

**Fig. 10:**
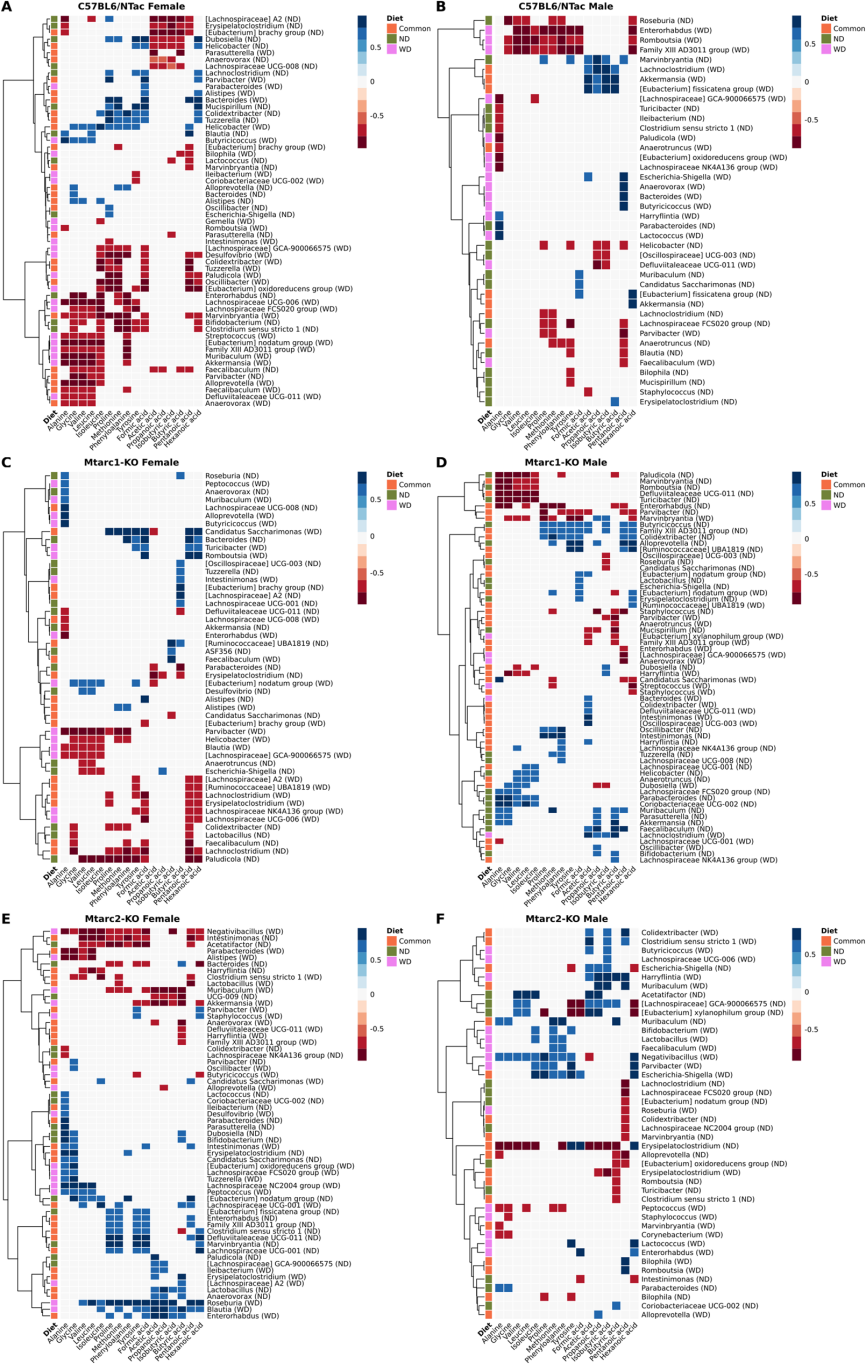
Correlation coefficients between bacterial taxa and amino acids (AAs) and short chain fatty acids (SCFAs) in C57BL6/NTac, Mtarc1-KO, and Mtarc2-KO female and male mice groups fed WD and the control ND/WD diets. Orange squares indicate correlations common to both diets, green squares are unique to the ND diet, and pink squares are unique to the WD diet

Next, we calculated the Spearman coefficient correlations between gut bacterial genera and stool metabolites (AAs and SCFAs) (Supplementary Tabele 3), In total, we obtained 633 correlations between 64 bacterial taxa and at least one metabolite in at least one study group with the magnitude of value higher than 0.6, as visualized in heatmaps (Figs. 9 and 10). Although the number of correlations across individual taxa was highly variable and depended on the mouse strain, sex, and diet, several taxa showed directionally consistent associations in multiple metabolites and contexts.

Of the bacteria with consistent negative associations with metabolites under GAN, *Akkermansia*, *Bifidobacterium*, *UCG-003* and *Family XIII*/*Clostridium sensu stricto*/*Lachnospiraceae* UCG-008 strongly correlated with multiple AAs (e.g., Glycine, Valine, Leucine, Isoleucine, Methionine, Phenyloalanine, Tyrosine) in Mtarc1-KO males, suggesting a cluster of Firmicutes linked with lower AAs levels. Of the bacteria with strong negative associations with SCFAs in GAN-fed mice, *Akkermansia* was negatively correlated with multiple SCFAs in B6 females, *Roseburia* with multiple SCFAs in Mtarc1-KO females, and *Butyricicoccus* with butyric acid in C57BL/6NTac males. *Lachnospiraceae*/*Lachnoclostridium* exhibited mixed associations with certain AAs, displaying both positive and complex signals with SCFAs across various contexts in male Mtarc1-KO and C57BL/6NTac female. In C57BL/6NTac female GAN feeding, *Lachnoclostridium* showed positive associations with several SCFAs, highlighting strain/sex specificity. Of the taxa that showed strong positive signals to GAN, *Anaerotruncus* was positively correlated with a block of methionine/phenylalanine/tyrosine and some SCFAs in Mtarc1-KO males, *Bilophila* with multiple AAs and butyric acid in Mtarc1-KO males, *Faecalibaculum* and *Blautia* with AAs in C57BL/6NTac females and Mtarc1-KO males, respectively. *Paludicola*, *Enterorhabdus*, *Enterorhabdus*, and *Dubosiella* also formed strong blocks of negative correlations in GAN-fed Mtarc1-KOmales.

WD-feeding resulted in many large-magnitude correlations (often *r* ≈ 0.70–0.96) involving both AAs and SCFAs, with multiple taxa showing directionally consistent blocks across related metabolites and repeated across all mouse strains. Of the genera with broad negative associations with AAs under WD, *Akkermansia* showed strong negative correlations with multiple AAs in C57BL/6NTac male and Mtarc2-KO female. *Alistipes* showed a consistent negative block of correlations with various AAs (approximately − 0.89 to − 0.75) in Mtarc2-KO females. *Alloprevotella* exhibited very strong negative associations with a wide amino-acid block (up to − 0.96), one of the most pronounced negative panels in the WD-fed mice. Both the Family XIII AD3011 group (C57BL/6NTac mice) and *Marvinbryantia* (C57BL/6NTac females) showed repeated strong negative correlations with several AAs. In turn, strong positive associations with multiple AAs showed *Bacteroides* in C57BL/6NTac females, *Intestinimonas* in Mtarc2-KO females, and *Negativibacillus* in Mtarc2-KOmales. SCFAs correlations patterns under WD revealed multiple strong negative associations with *Oscillibacter* and *Desulfovibrio* in C57BL/6NTac females, and with *Erysipelatoclostridium* in Mtarc2-KO males. *Roseburia* was positively associated with several SCFAs, and *Desulfovibrio* was positively associated with acetic acid in Mtarc2-KO females.

## Discussion

There is no single specific type of Western diet; the term describes a broad dietary pattern characterized by high consumption of processed foods, red and processed meats, refined grains, high-sugar drinks, and saturated and trans fats, with low intake of fruits, vegetables, and fiber. However, the specific foods and quantities can vary significantly across different regions and even within individual households, and access to healthy food options can be influenced by socioeconomic status. Therefore, preclinical studies using laboratory animals allow for better diet standardization.

We compared the body mass gain and modifications of the gut microbial community, as well as fecal abundance of SCFAs and AAs, within Mtarc1-KO, Mtarc2-KO and C57BL6/NTac background male and female mice, fed for 16 weeks with a high-fat/high-sucrose diet (WD), a high-fat/high-fructose/high-cholesterol diet (GAN) and the corresponding control normal diets (ND/WD or ND/GAN), purchased from the Research Diet. While both diets have been used in preclinical research to induce obesity, insulin resistance, hepatic steatosis, and inflammation, the GAN diet is considered more obesogenic compared to other diet-induced obesity models. The GAN diet model is also highly translatable to humans, as it mimics the progression and characteristics of metabolic dysfunction-associated steatohepatitis (MASH) in mice [30–32].

The weight gain of all mouse groups fed the control ND/WD was similar, and only in Mtarc2-KO females and males fed the control ND/GAN the weight gain was significantly lower than in the two other mouse strains. Feeding with WD and GAN significantly increased the body weight of all groups of mice, except for female Mtarc2-KO and Mtarc1-KO mice, respectively, compared to mice fed the corresponding ND diets. Feeding with NDs resulted in significantly greater weight gain in males of all three strains and female Mtarc1-KO and C57BL6/NTac mice fed ND/GAN than in those fed ND/WD, whereas feeding with GAN resulted in higher body weight gain only in Mtarc2-KO females compared to feeding with WD (Fig. 2C and D).

The differences in body weight gain patterns were associated with changes in the gut microbiota profiles. The GAN feeding decreased α-diversity in all mouse groups, except for Mtarc 1-KO females, compared to the corresponding groups of mice fed the control ND/GAN diet. A WD diet reduced the Shannon index in C57BL/6 N females and males, and in Mtarc 2-KO females, and decreased the Chao1 index in C57BL/6 N females and males, and in Mtarc1-KO females, when compared with mice fed a ND/WD. The β-diversity of the microbial community was altered by both GAN and WD in all groups studied. For each of the mouse strains tested, the difference in the number of differentially abundant taxa in the GAN-fed groups of mice and their corresponding controls was higher than that between mice fed a WD and their corresponding controls.

As shown previously, GAN feeding reduced the diversity of the gut microbiota, altered the composition of the gut microbiota, and decreased the abundance of SCFAs [33]. Representative members of the *Lachnospiraceae* and *Ruminococcaceae* families are thought to produce SCFAs [34]. Among the genera belonging to the *Lachnospiraceae* family, we identified seven and nine as being over-represented and under-represented, respectively, in at least one GAN-fed group of mice. For the groups of WD-fed mice, six genera showed a direction of change consistent with those observed in GAN-fed mice, whereas five showed the opposite direction of change. The GAN diet selectivity increased the relative abundance of three genera within the *Ruminococcaceae* family. A question remains about the potential links between high fructose intake and the accompanying metabolic consequences [35] in relation to the composition of the gut microbiota. Here, we observed both an increase and a decrease in the relative levels of specific taxa, which may be related to both a GAN and a WD diet. When we compared the final amount of weight gained in mice fed the control ND/WD and ND/GAN diets, we found significantly greater weight gain in males of all three strains and female Mtarc1-KO and C57BL6/NTac mice fed ND/GAN than ND/WD. (Figure 2A and B). In turn, feeding with GAN resulted in higher body weight gain only in Mtarc2-KO females than in those fed with WD (Fig. 2C and D). It is generally believed that changes in the intestinal microbiota may affect metabolic functions of a host [36]. SCFAs are the richest fecal metabolites generated by intestinal bacteria, such as *Faecalibacterium prausnitzii*,* Roseburia intestinalis*, and *Anaerostipes butyraticus*, which maintain intestinal homeostasis.

Data from seven SCFAs analyzed in fecal sample extracts at the end of the experiment revealed differences in the relative abundance of only three SCFAs. Acetic acid levels increased in Mtarc1-KO females fed GAN, Mtarc1-KO males fed WD and GAN, and Mtarc2-KO males fed WD compared with the same strains fed the corresponding control ND, whereas formic acid levels increased in C57BL/6NTac females fed GAN, and decreased in GAN-fed Mtarc2-KO females. Similar comparisons conducted on AAs data also revealed some differences in their relative abundance between strains, sex and diets, but again, there was no particular pattern.

Acetate, mainly produced by *Bifidobacteria spp*., maintains the epithelium barrier and regulates intestinal inflammation, butyrate is an essential source of energy for the colonocytes [37, 38], and formic acid is considered a mediator of metabolic interactions between mammals, diet, and the gut microbiome [39–41]. Bacterial metabolism in the colon is also related to the availability of AAs [42], although the role and potential mechanisms of AAs action in the distal colon are not clear. Our study revealed several correlations between abundances of bacterial taxa and the fecal metabolites, and the most frequent taxa with strong correlations were represented by *Enterorhabdus*,* Erysipelatoclostridium*,* Marvinbryantia*,* Parvibacter*,* Family XIII AD3011 group*,* Muribaculum*,* Paludicola*,* Helicobacter*,* Akkermansia*,* and Intestinimonas*. Many taxa demonstrated strong negative correlations with SCFAs (e.g., butyric, acetic, propanoic acids) under high-fat diets, particularly *Alloprevotella*,* Lachnospiraceae UCG-006*,* Muribaculum*, and *Marvinbryantia*. These SFCA producers are typically linked to leanness and metabolic health [43, 44], while a strong negative correlation with increased bacterial abundance and decreased metabolite level, or vice versa, suggests either decoupling between taxon abundance and measured luminal concentration due to absorption/location effects, or diet-driven reprogramming where taxa presence does not translate into higher stool SCFAs. Elevated branched-chain AAs in stool and circulation are strongly associated with obesity and insulin resistance, and strong negative correlations with some branched-chain and aromatic AAs may mark taxa that are depleted in obesity, thus failing to metabolize excess dietary AAs. However, stool metabolite directions reflect luminal pools, not serum, so the functional interpretation requires care. Nevertheless, our study may support the consensus that Western Diets promote dysbiosis, associating with and metabolic disorder, partly by restructuring the metabolic capacity of the microbiome, that is characterized by falling of SCFAs production and rising of gut permeability and inflammation [45].

In humans, expression of *MTARC1* is highest in white adipose tissue, thyroid, breast, and liver, whereas that of *MTARC2* is highest in the liver [46]. In male mice, *Mtarc1* mRNA is expressed almost exclusively in the liver, whereas *Mtarc2* is expressed in the small intestine, liver, and kidney [46]. Nutritional state regulates expression of Mtarc in both humans and mice [4, 47, 48]. Expression of the Mtarc1 and Mtarc2 proteins, as well as their N-reductive activity, in murine liver falls under fasting conditions, and increases after exposure to an HFD [4]. Although human GWAS [6–9, 49–53] and an exome-wide association study [46] identified several variants of *MTARC1*, including missense variants p.Ala165Thr and p.Met187Lys, as being associated with lower hepatic fat, reduction in liver enzymes, and protection against most causes of cirrhosis, a study by Smarings et al. [46] showed that whole-body Mtarc1 deficiency caused those mice vulnerable to accumulation of liver triglycerides, liver inflammation, or fibrosis. Three independent studies showed that hepatocyte-specific knockdown of *Mtarc1* (GalNAc*-siMtarc1*) in mice with GAN diet-induced obesity and metabolic dysfunctions improved liver triglycerides, enzymes, and histopathology [9, 10, 54], despite differences in the duration of the experiments (6, 7, or 44 weeks) and the timing of GalNAc*-siMtarc1* administration. This suggests that whole-body KO models do not capture the liver-specific role of Mtarc1.

Sex plays an essential role in weight gain and the metabolic consequences of diet in rodents [55]. Specifically, male and female rodents often exhibit distinct patterns of weight gain, fat accumulation, and metabolic adaptations when exposed to obesogenic diets. These differences, observed also within species, highlight the complex interplay of sex hormones, genetics, and environmental factors. Male C57BL/6 mice typically reach an obese state more rapidly than female mice, which exhibit greater resistance to diet-induced obesity and better maintenance of glucose homeostasis compared to males [56]. Male rodents on HFDs accumulate more visceral fat and brown adipose tissue, which is responsible for dissipating energy through heat generation, compared to females [56–64]. It is considered that estrogen in female rodents plays a protective role in metabolic health, and sex dependent differences in gene expression patterns in adipose tissue and liver as well as in gut microbiome composition, are influenced by sex hormones [65–68]. Therefore, sex must be considered as an essential biological variable in studies of obesity and metabolic diseases. In our mouse models of diet-induced obesity, both Western-type diets caused an increase in body weight in male mice compared with females, and the weight gain was significantly less in Mtarc1-KO and Mtarc2-KO mice than in C57BL/6 N mice.

The primary fat source in the WD was butter, whereas that in the GAN was palm oil, with added soybean oil and lard. The main carbohydrate source in the WD was sucrose, whereas that in GAN was fructose. GAN also contained 2% cholesterol, whereas the WD contained 0.1% cholesterol. Despite significant differences in diet composition, both diets are obesogenic. Our data reveal notable differences in the effects of an HFD enriched in fructose compared to a high-fat/high-sucrose diet, as well as the impact of the two control diets. The ND/WD diet contained one-quarter the amount of butter found in the WD diet, while the ND/GAN contained only soybean oil and lard (at the exact amounts as in the GAN). The primary carbohydrate source in ND/WD was corn starch, whereas that in ND/GAN was corn starch and (to a lesser extent) dextrose and sucrose. Each of these dietary ingredients alone may be a potent regulator of body weight, as well as microbial and metabolite abundance.

The study on the impact of American diet and Mediterranean, Japanese, and Maasai/ketogenic diets on metabolic health across four genetically distinct inbred mice demonstrated that individualized diet strategies improve health outcomes in mice that were highly dependent on genetic background [69, 70]. The study on C57BL/6J and FVB/NJ mouse strains has shown that the integration of genetics, intestinal microbiota variation, and sex may predict response to American and ketogenic diets, which are high in fat and vary in carbohydrate composition [42]. Our study partly confirmed these earlier observations.

We recently reported [71] that Mtarc2-KO mice were more resistant to diet-induced obesity and related metabolic challenges compared to WT mice, which again highlighted a potential role of the Mtarc complex in regulating lipid metabolism in mice. A novel aspect of the current study was the comparison of the effects of two obesogenic diets on weight gain and the composition of the intestinal microbiome in both Mtarc1-KO and Mtarc2-KO mouse strains. However, our study also has limitations, including a relatively small sample size of groups. Like in our previous preclinical studies [5, 22, 71–73], the number of animals used (8–10 mice per group) was allowed and is under the prospective scrutiny of the Local Ethics Committee for Animal Experiments. Furthermore, although our cross-sectional study allowed assessing the prevalence, distribution, and potential associations of the body weight gain and changes of the intestinal microbiota within mice groups at a specific point in time, we could not establish cause-and-effect relationships and could not translate the results of the standarized animal experiment to human populations whose compositions of the gut microbiome varies geographically, and due to combination of genetics and environmental factors such as lifestyle, hygiene, local food preparation methods, social behaviors, and others.

## Conclusion

We confirmed that differences in the body weight gain, as well as changes in the composition of the gut microbiome and metabolome, between a background mouse strain and Mtarc1-KO or Mtarc2-KO mice were affected by sex and diet. As a consequence, preclinical studies on animal models of obesity need to consider not only the selection of the appropriate mouse strain, but also the sex used and the composition of the study and control diets. Each of these factors may significantly impact the outcomes and interpretations of research, as we show clearly herein. However, since the final effects of the diet are likely dependent on a complex combination of specific ingredients, further studies are needed to gain a more comprehensive understanding of the interrelationship between obesity and intestinal microbial dysbiosis.

## Supplementary Information

Supplementary Material 1.

Supplementary Material 2.

Supplementary Material 3.

Supplementary Material 4.

Supplementary Material 5.

## Data Availability

The datasets presented in this study can be found in online repositories. The names of the repository and accession number can be found below: https://www.ncbi.nlm.nih.gov/BioProject/PRJNA1260802.
