# Supplementary figures and images for: Sex- and mouse strain-related differences in body weight gain, composition of the gut microbiota, and levels of selected metabolites in response to a Western-style diet
Source: BMC Gastroenterol. 2026 Feb 4;26:158. doi: 10.1186/s12876-026-04647-2 (PMC12964887; doi:10.1186/s12876-026-04647-2)

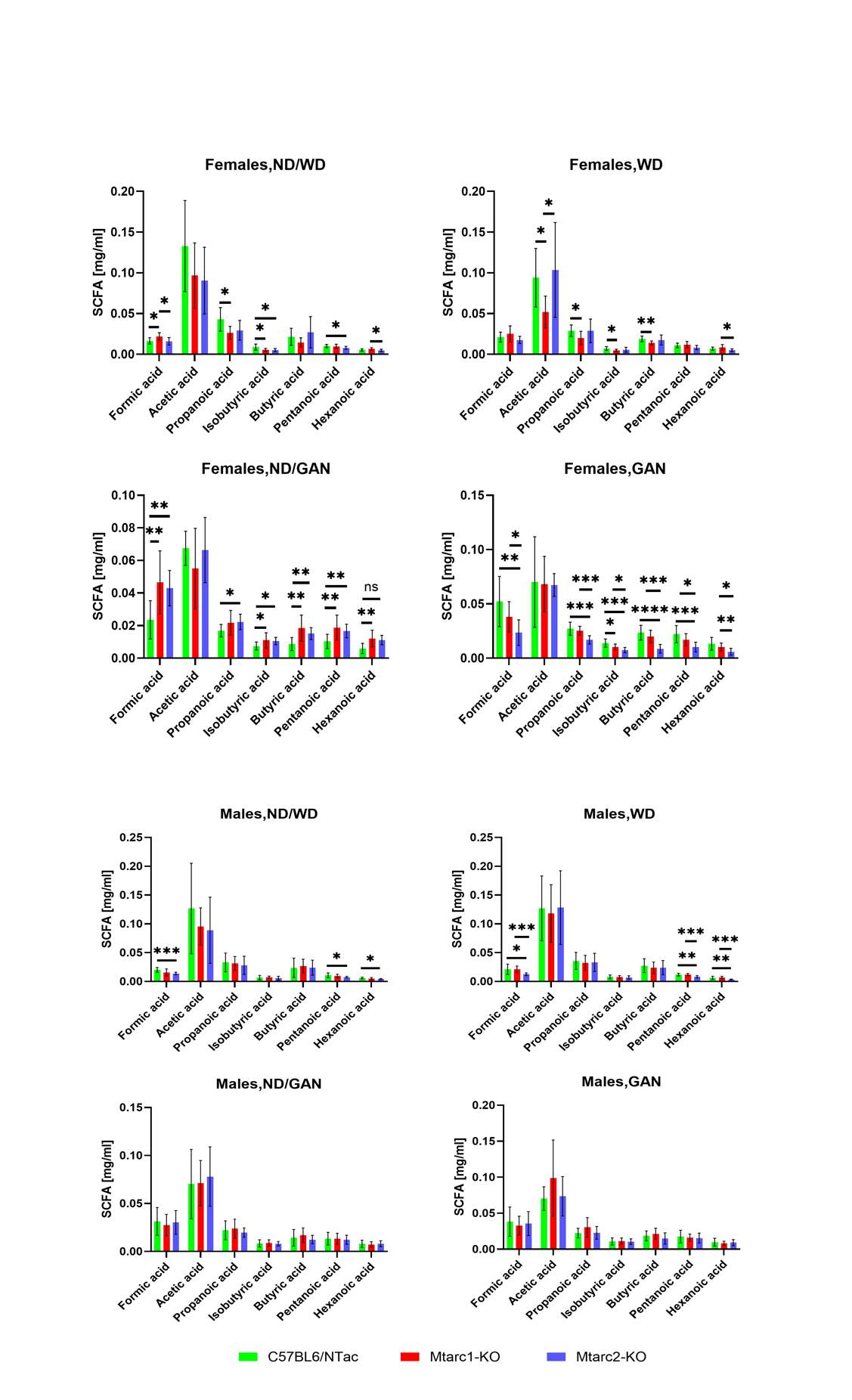

Supplement: Supplementary file 1 — Supplementary Material 1. [file 12876_2026_4647_MOESM1_ESM.png]

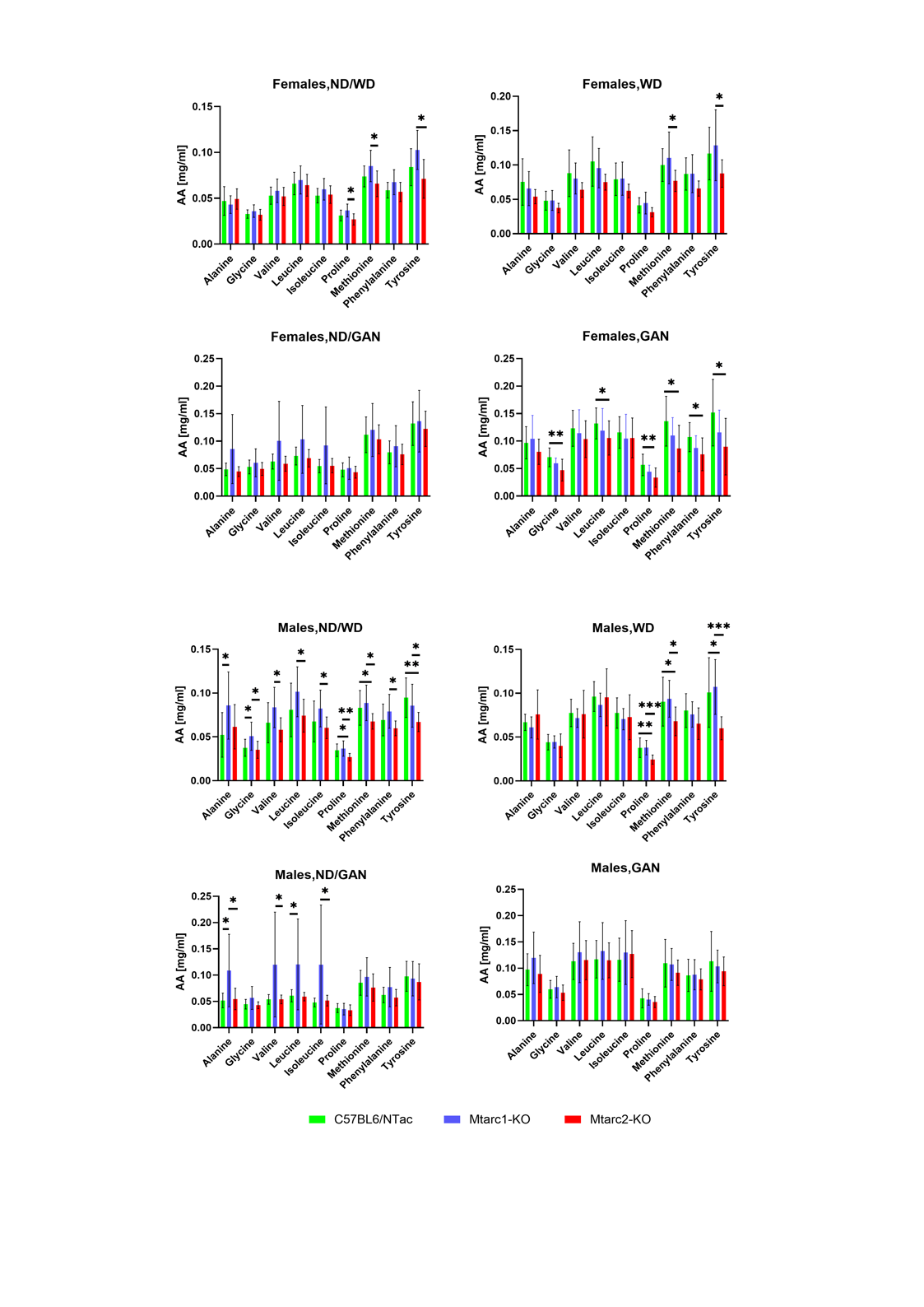

Supplement: Supplementary file 2 — Supplementary Material 2. [file 12876_2026_4647_MOESM2_ESM.png]
